# Supplementary material for: Bile proteome reveals biliary regeneration during normothermic preservation of human donor livers
Source: Nat Commun. 2023 Nov 30;14:7880. doi: 10.1038/s41467-023-43368-y (PMC10689461; doi:10.1038/s41467-023-43368-y)
Supplement: Supplementary file 1 — Supplementary Information [file 41467_2023_43368_MOESM1_ESM.pdf]

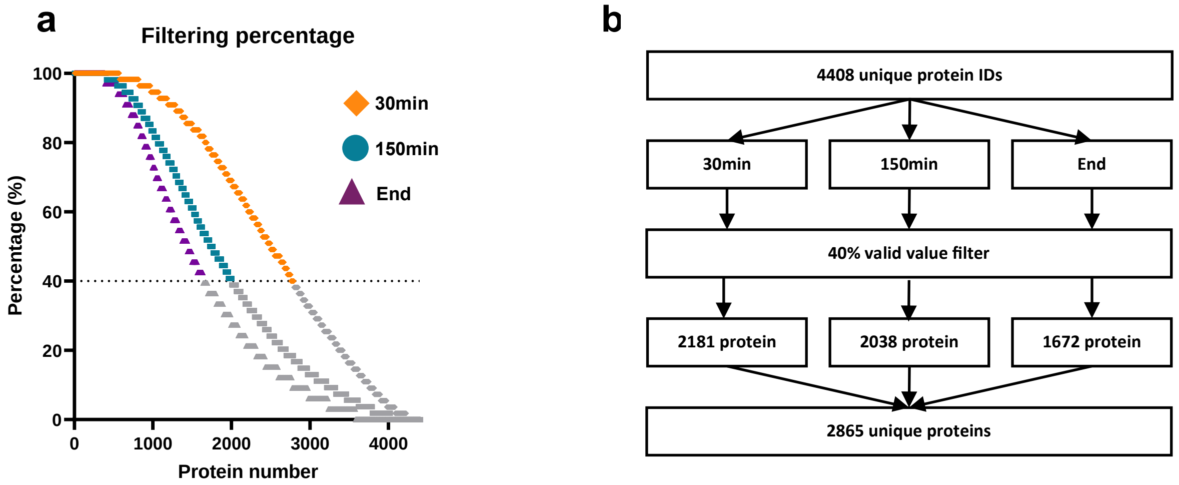

Figure S1. Graphic depicting protein filtering at each time point. (**A**) representation of individual proteins at each time point (30min, 150min, End) and their corresponding cut offs with 40% valid value filter. (**B**) flow chart showing total, filtered and final protein ID counts after 40% valid value filtering.

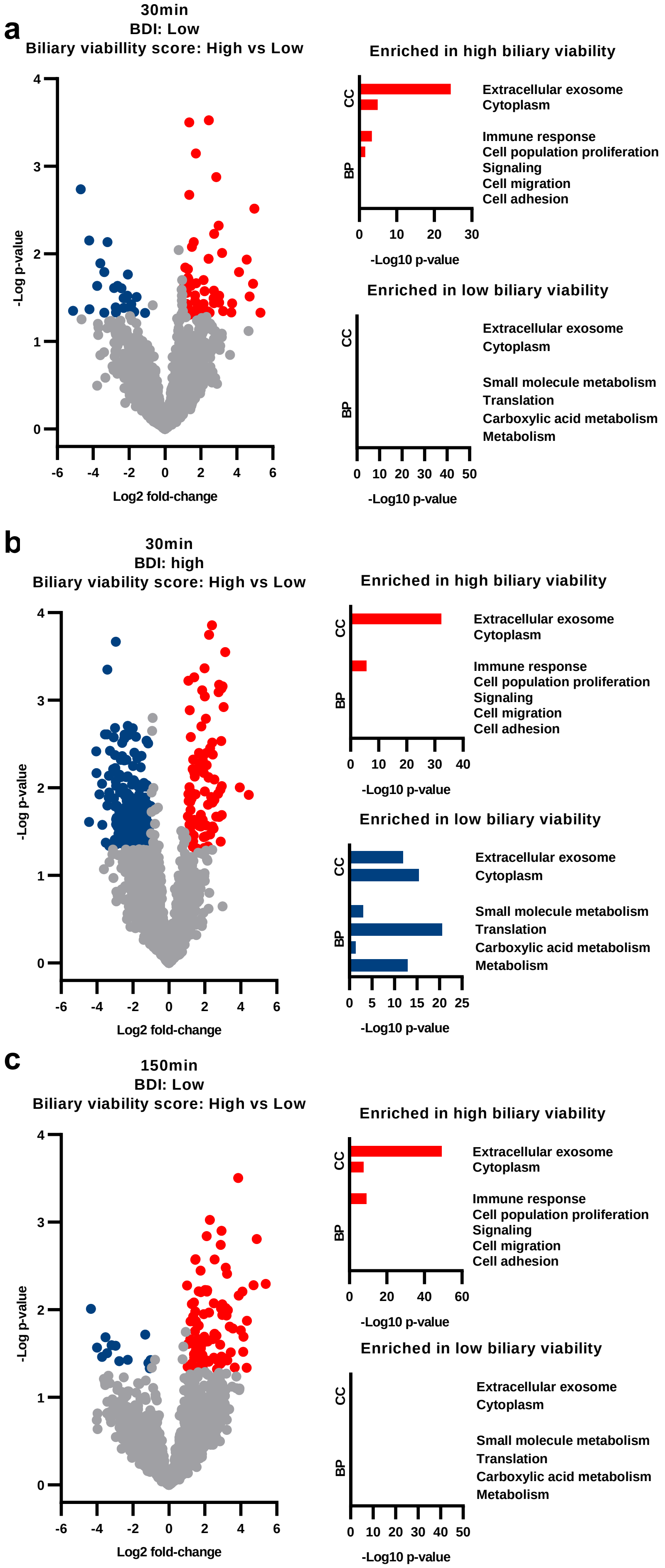
a
30min
BDI: Low
Biliary viabillity score: High vs Low
-Log p-value
Log2 fold-change
Enriched in high biliary viability
CC
BP
Extracellular exosome
Cytoplasm
Immune response
Cell population proliferation
Signaling
Cell migration
Cell adhesion
-Log10 p-value
Enriched in low biliary viability
Extracellular exosome
Cytoplasm
Small molecule metabolism
Translation
Carboxylic acid metabolism
Metabolism
-Log10 p-value
b
30min
BDI: high
Biliary viability score: High vs Low
-Log p-value
Log2 fold-change
Enriched in high biliary viability
Extracellular exosome
Cytoplasm
Immune response
Cell population proliferation
Signaling
Cell migration
Cell adhesion
-Log10 p-value
Enriched in low biliary viability
Extracellular exosome
Cytoplasm
Small molecule metabolism
Translation
Carboxylic acid metabolism
Metabolism
-Log10 p-value
c
150min
BDI: Low
Biliary viability score: High vs Low
-Log p-value
Log2 fold-change
Enriched in high biliary viability
Extracellular exosome
Cytoplasm
Immune response
Cell population proliferation
Signaling
Cell migration
Cell adhesion
-Log10 p-value
Enriched in low biliary viability
Extracellular exosome
Cytoplasm
Small molecule metabolism
Translation
Carboxylic acid metabolism
Metabolism
-Log10 p-value

Figure S2. Protein abundance between livers with high and low bile duct injury, and high and low biliary viability score. Volcano plots showing significance and fold-change of protein intensity, comparing livers with high and low biliary viability scores within the (**A**) low BDI group at 30min, (**B**) high BDI group at 30min and (**C**) low BDI group at 150min. Significant proteins ($p<0.05$, >2-fold change) are highlighted red for upregulated (enriched in high biliary viability score livers) and blue for downregulated (enriched in low biliary viability score livers). Statistics were performed using a two-tailed Students t-test with a permutation-based FDR of 0.05 to assess multiple comparisons. Cellular component (CC) and biological process (BP) gene ontology (GO) pathways are displayed as –Log10 p-value.

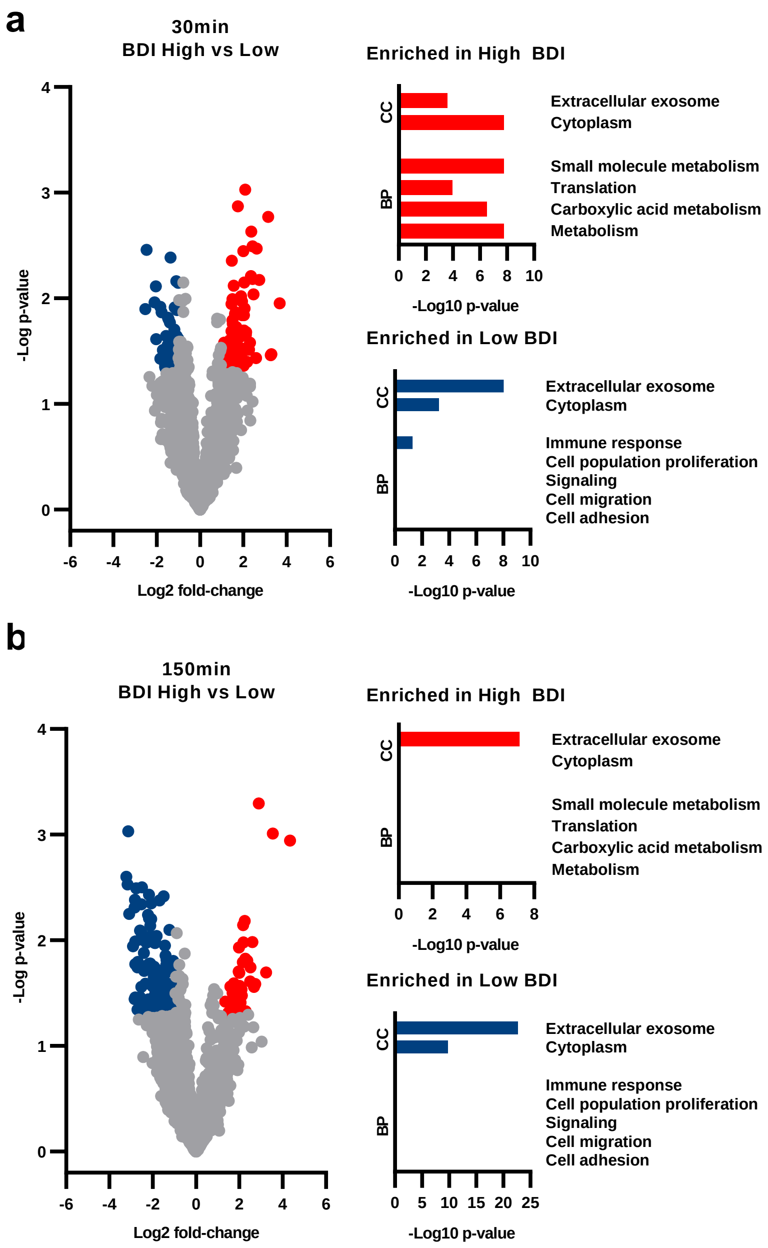

Figure S3. Protein abundance between livers with high and low BDI score. Volcano plots showing significance and fold-change of protein intensity, comparing high and low BDI livers at (**A**) 30min and (**B**) 150min. Significant proteins ($p<0.05$, >2-fold change) are highlighted red for upregulated (enriched in high BDI livers) and blue for downregulated (enriched in low BDI livers). Statistics were performed using a two-tailed Students t-test with a permutation-based FDR of 0.05 to assess multiple comparisons. Cellular component (CC) and biological process (BP) gene ontology (GO) pathways are displayed as –Log10 p-value.

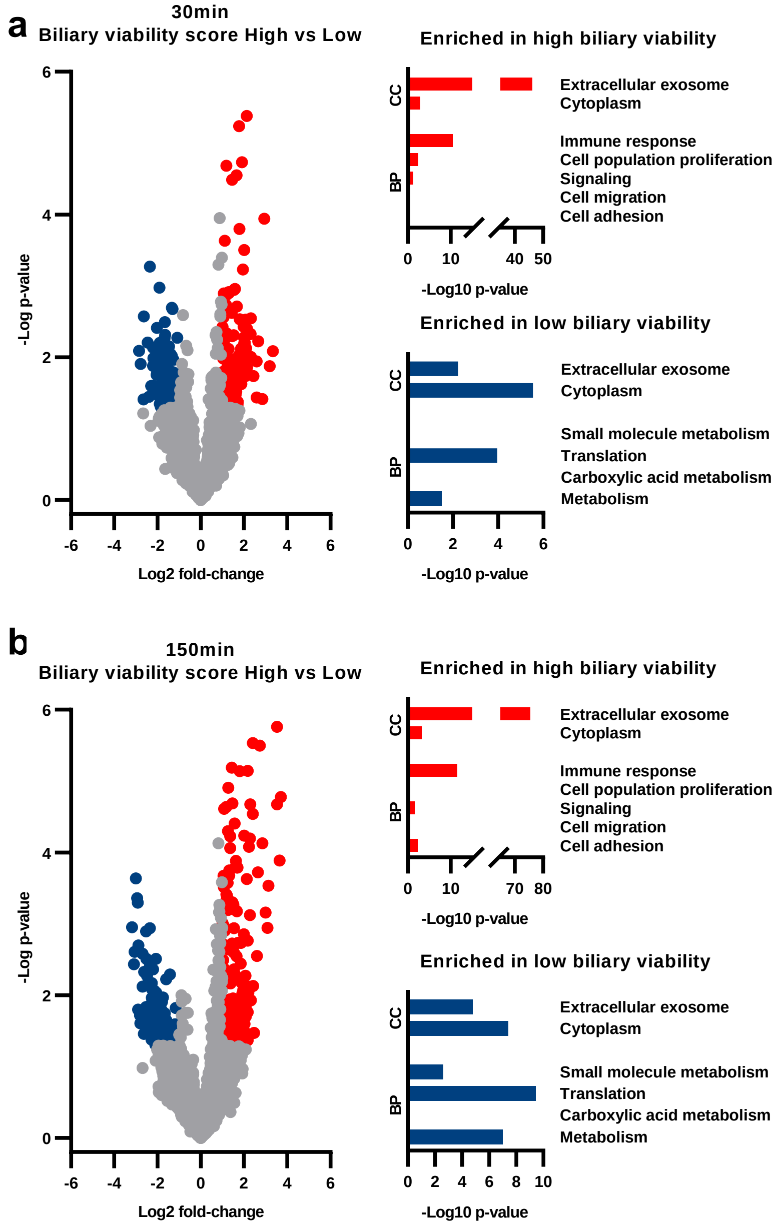

Figure S4. Protein abundance between livers with high and low biliary viability score. Volcano plots showing significance and fold-change of protein intensity, comparing high and low biliary viability score livers at (**A**) 30min and (**B**) 150min. Significant proteins ($p<0.05$, >2-fold change) are highlighted red for upregulated (enriched in high biliary viability score livers) and blue for downregulated (enriched in low biliary viability score livers). Statistics were performed using a two-tailed Students t-test with a permutation-based FDR of 0.05 to assess multiple comparisons. Cellular component (CC) and biological process (BP) gene ontology (GO) pathways are displayed as –Log10 p-value.

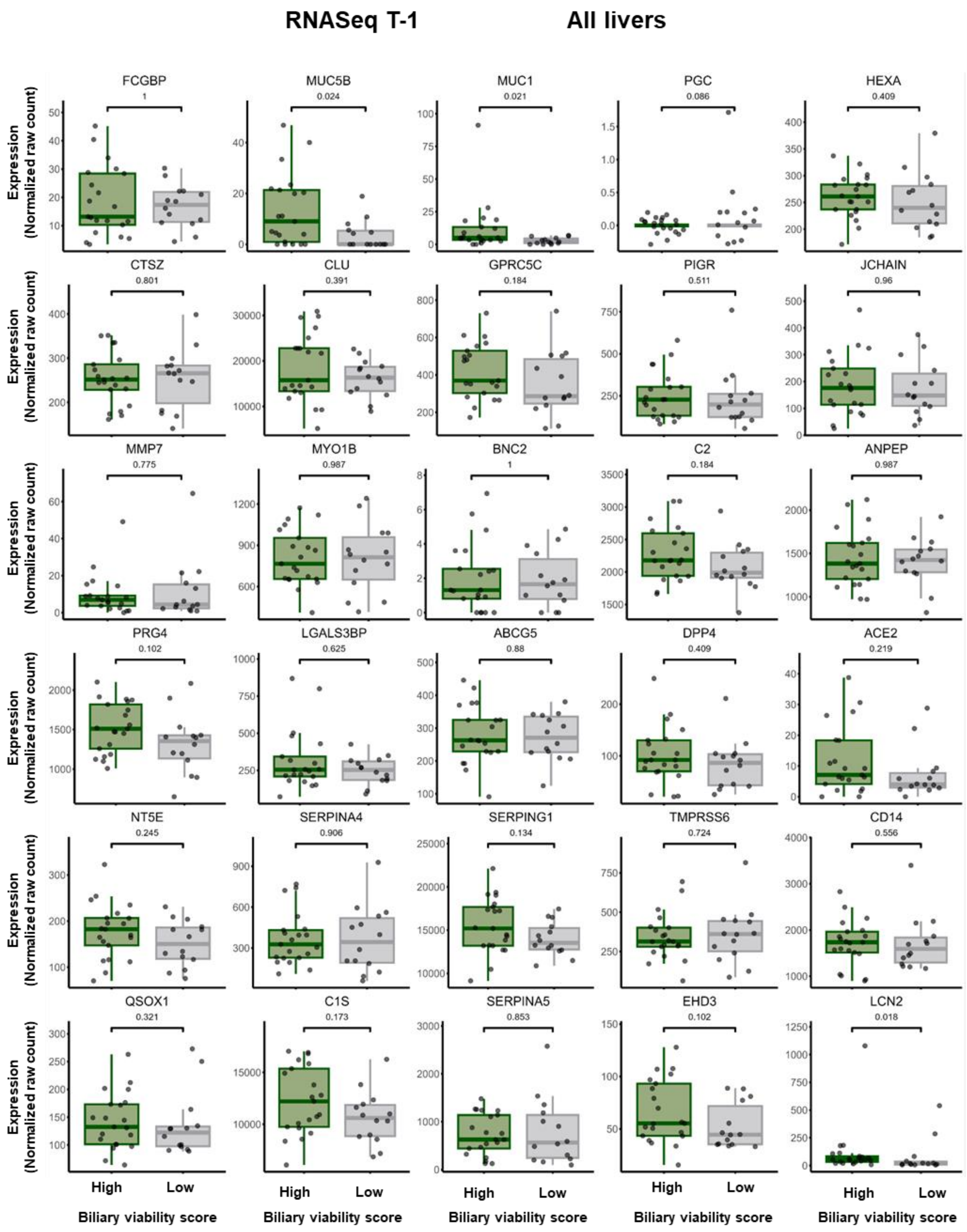

Figure S5. RNA sequencing of baseline liver tissue biopsies before machine perfusion in all livers. Data is presented as normalized raw counts. Groups are split into high (green) and low (grey) biliary viability scores based on previously mentioned criteria. Box plots display median as the center line, 25th and 75th percentiles, and minimum/maximum whiskers.

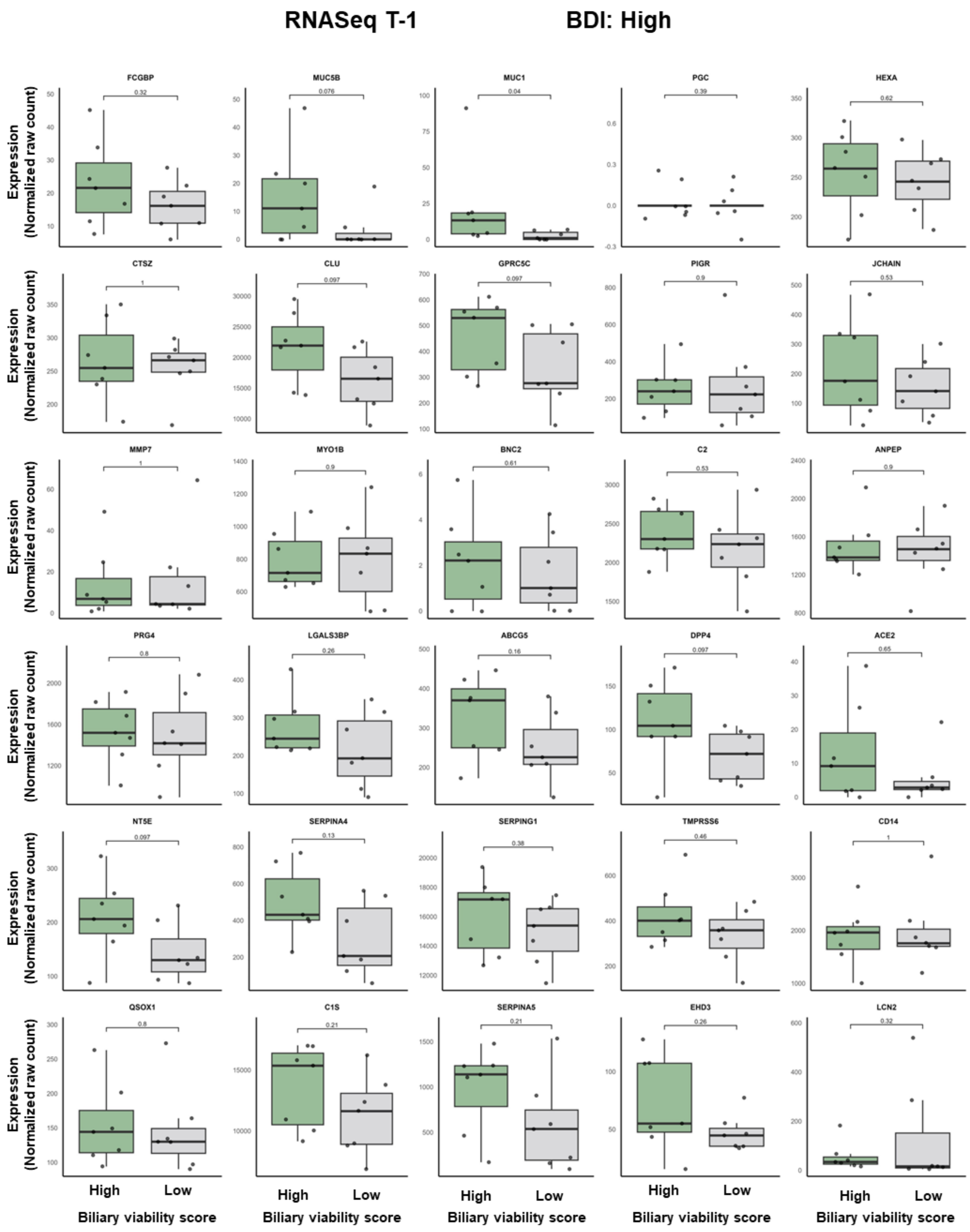

Figure S6. RNA sequencing of baseline liver tissue biopsies before machine perfusion in high BDI livers. Data is presented as normalized raw counts. Groups are split into high (green) and low (grey) biliary viability scores based on previously mentioned criteria. Box plots display median as the center line, 25th and 75th percentiles, and minimum/maximum whiskers.

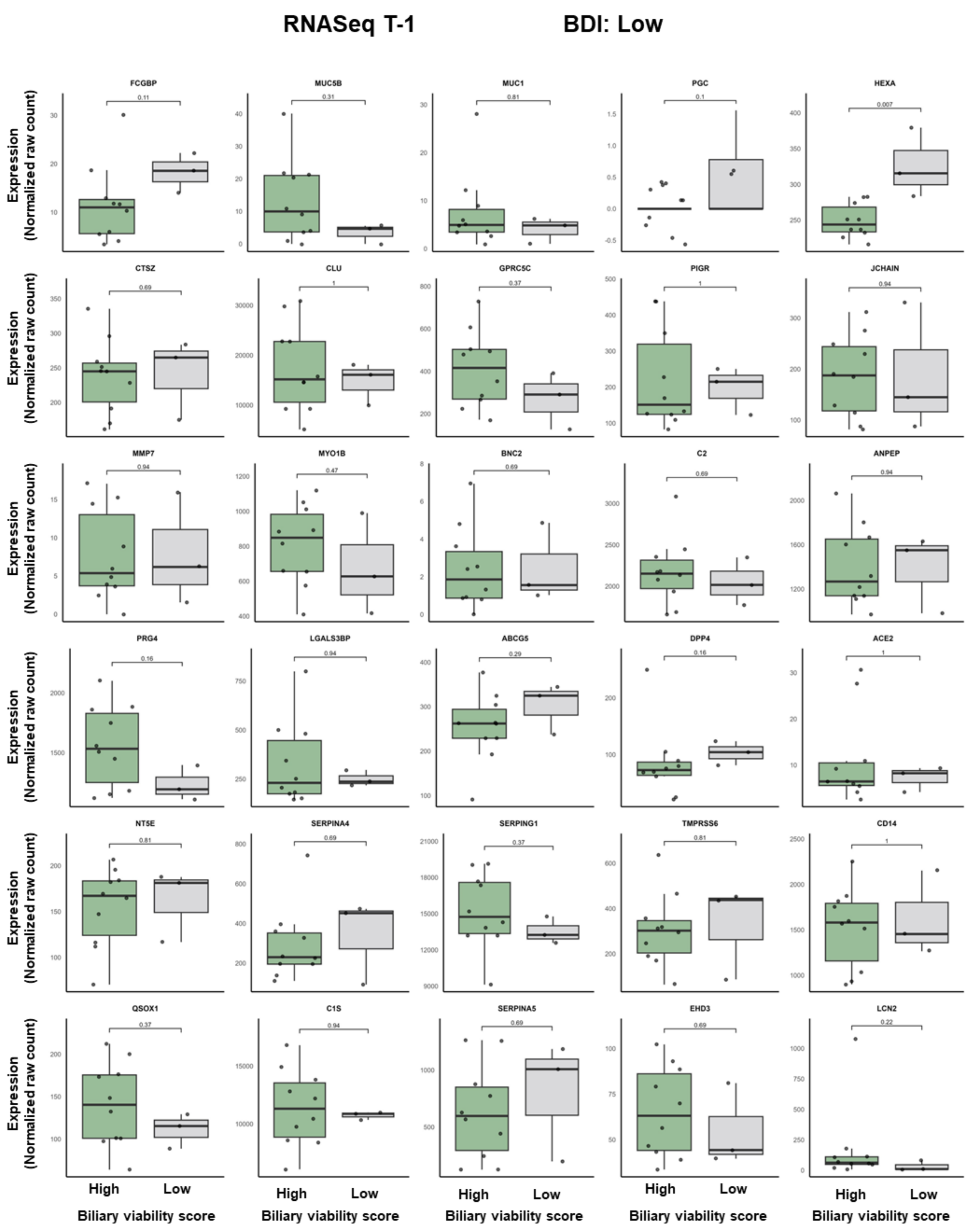

Figure S7. RNA sequencing of baseline liver tissue biopsies before machine perfusion in low BDI livers. Data is presented as normalized raw counts. Groups are split into high (green) and low (grey) biliary viability scores based on previously mentioned criteria. Box plots display median as the center line, 25th and 75th percentiles, and minimum/maximum whiskers.

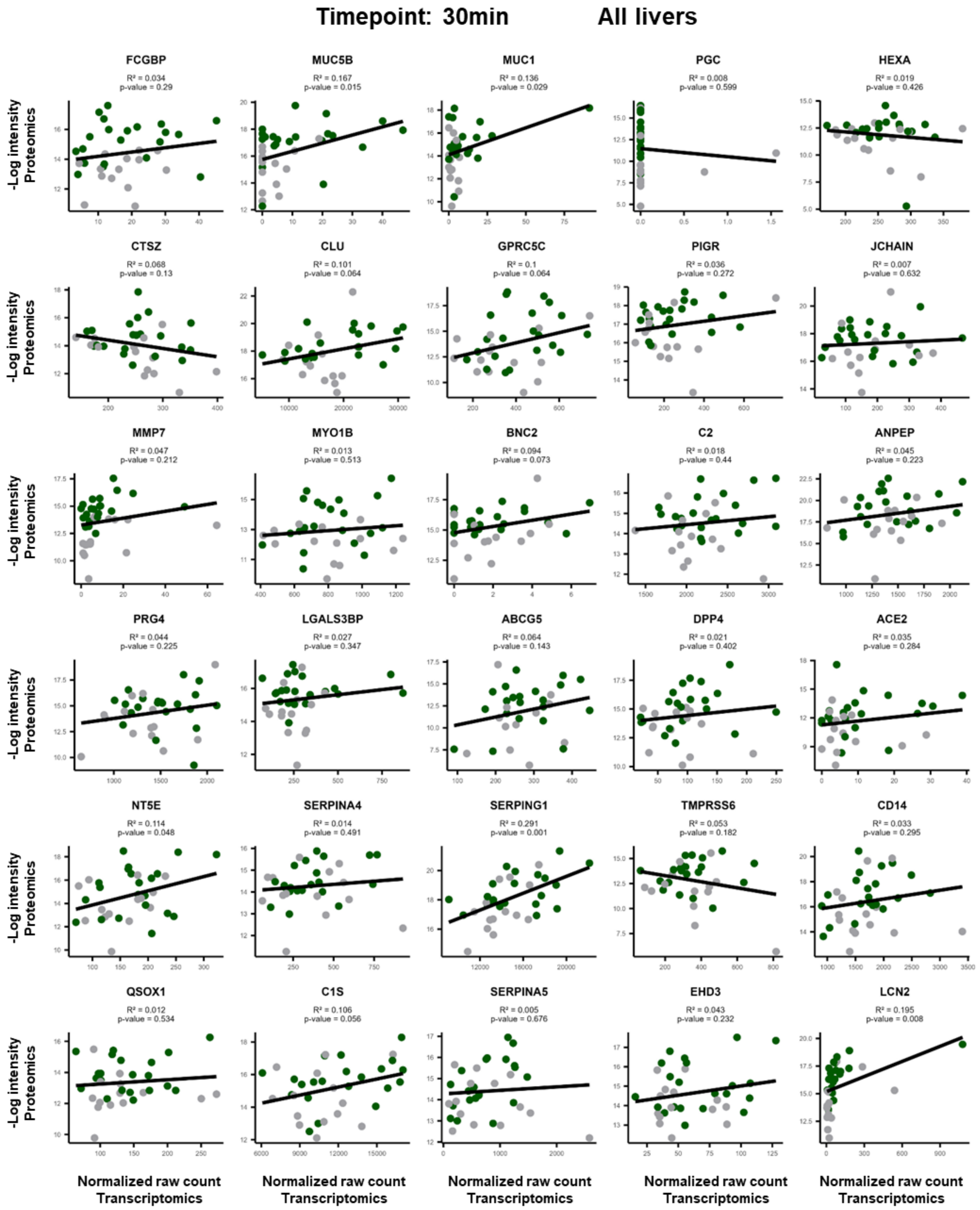

Figure S8. Correlation of RNA sequencing transcripts from baseline biopsies with bile protein intensity all livers at 30min. Data is presented as protein intensity (-Log transformed, y-axis) correlated with RNA sequencing transcripts (normalized raw counts, x-axis). R-squared values are displayed for

each plot. Correlation p-values were performed using simple linear regression. Livers with high biliary viability score are in green, low are in grey.

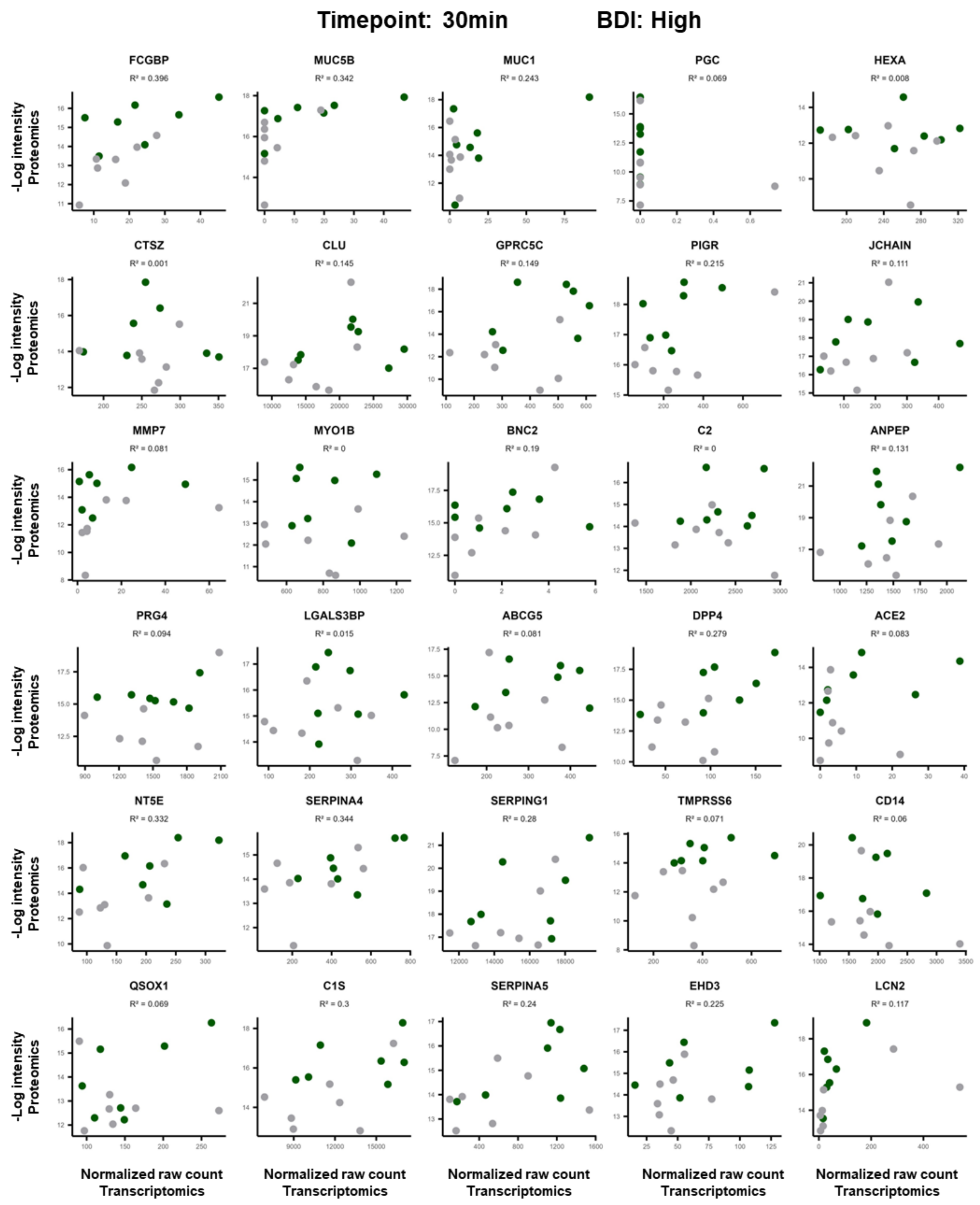

Figure S9. Correlation of RNA sequencing transcripts from baseline biopsies with bile protein intensity high BDI livers at 30min. Data is presented as protein intensity (-Log transformed, y-axis) correlated with RNA sequencing transcripts (normalized raw counts, x-axis). R-squared values are displayed for each plot. Livers with high biliary viability score are in green, low are in grey.

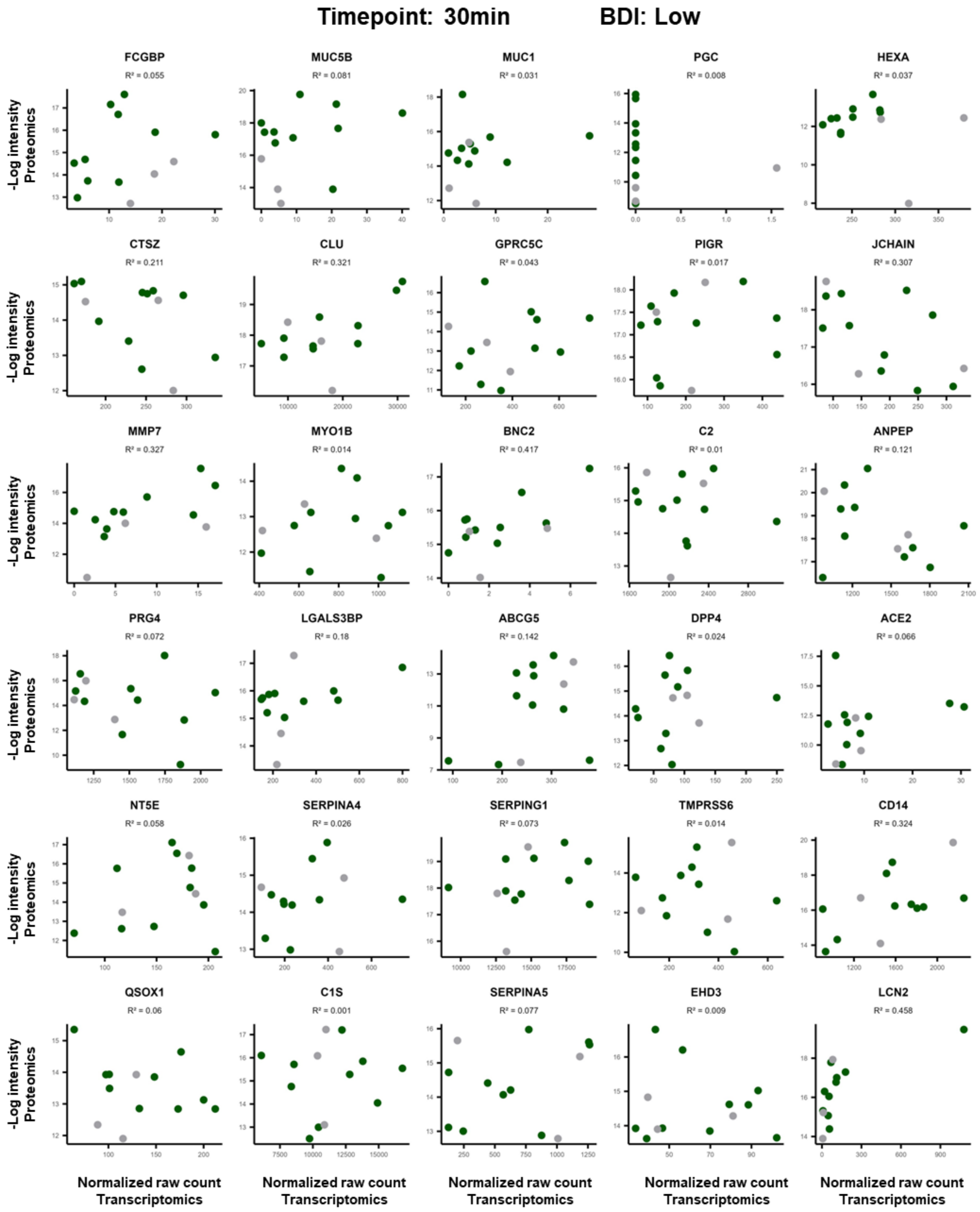

Figure S10. Correlation of RNA sequencing transcripts from baseline biopsies with bile protein intensity in low BDI livers at 30min. Data is presented as protein intensity (-Log transformed, y-axis) correlated with RNA sequencing transcripts (normalized raw counts, x-axis). R-squared values are displayed for each plot. Livers with high biliary viability score are in green, low are in grey.

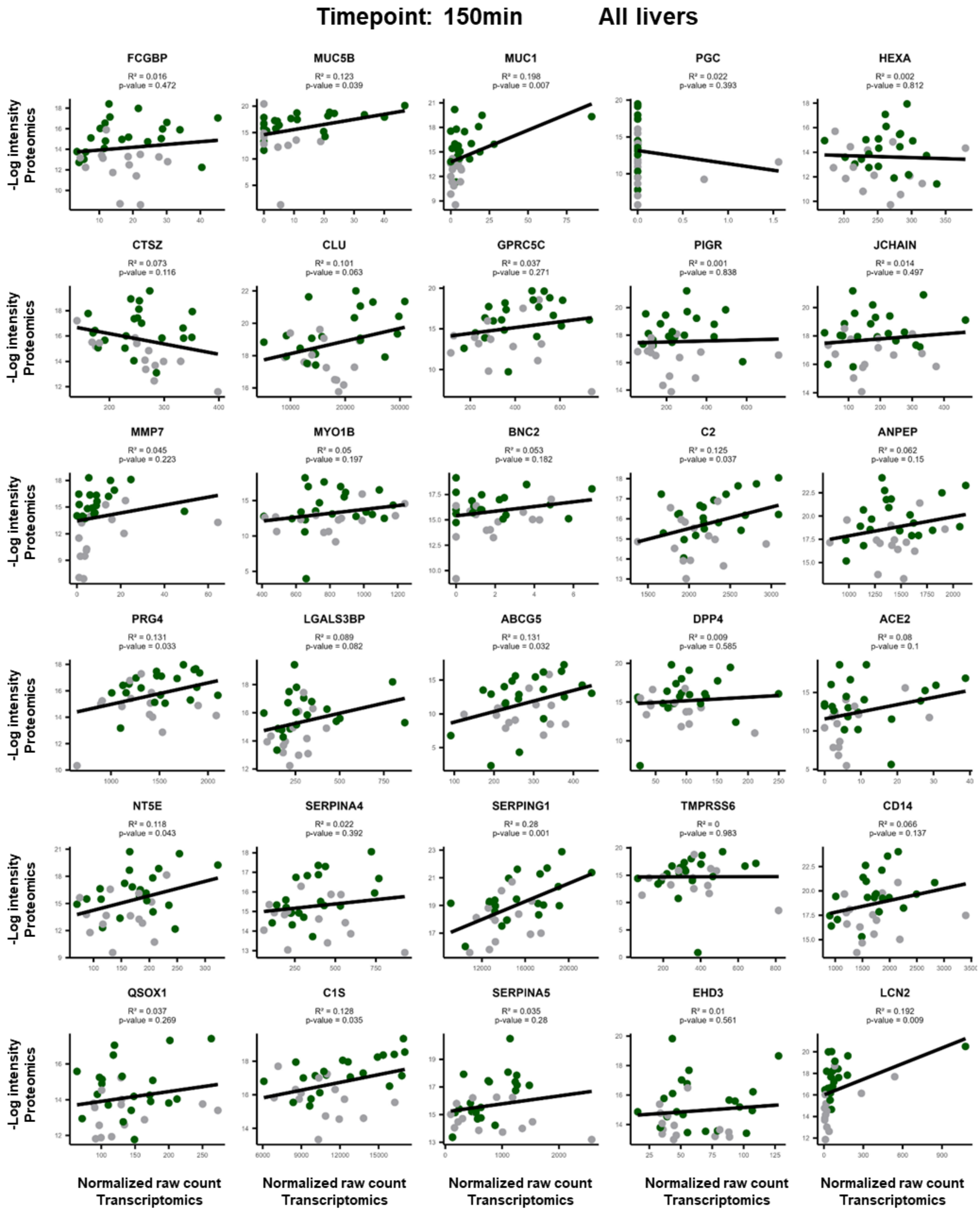

Figure S11. Correlation of RNA sequencing transcripts from baseline biopsies with bile protein intensity in all livers at 150min. Data is presented as protein intensity (-Log transformed, y-axis) correlated with RNA sequencing transcripts (normalized raw counts, x-axis). R-squared values are

displayed for each plot. Correlation p-values were performed using simple linear regression. Livers with high biliary viability score are in green, low are in grey.

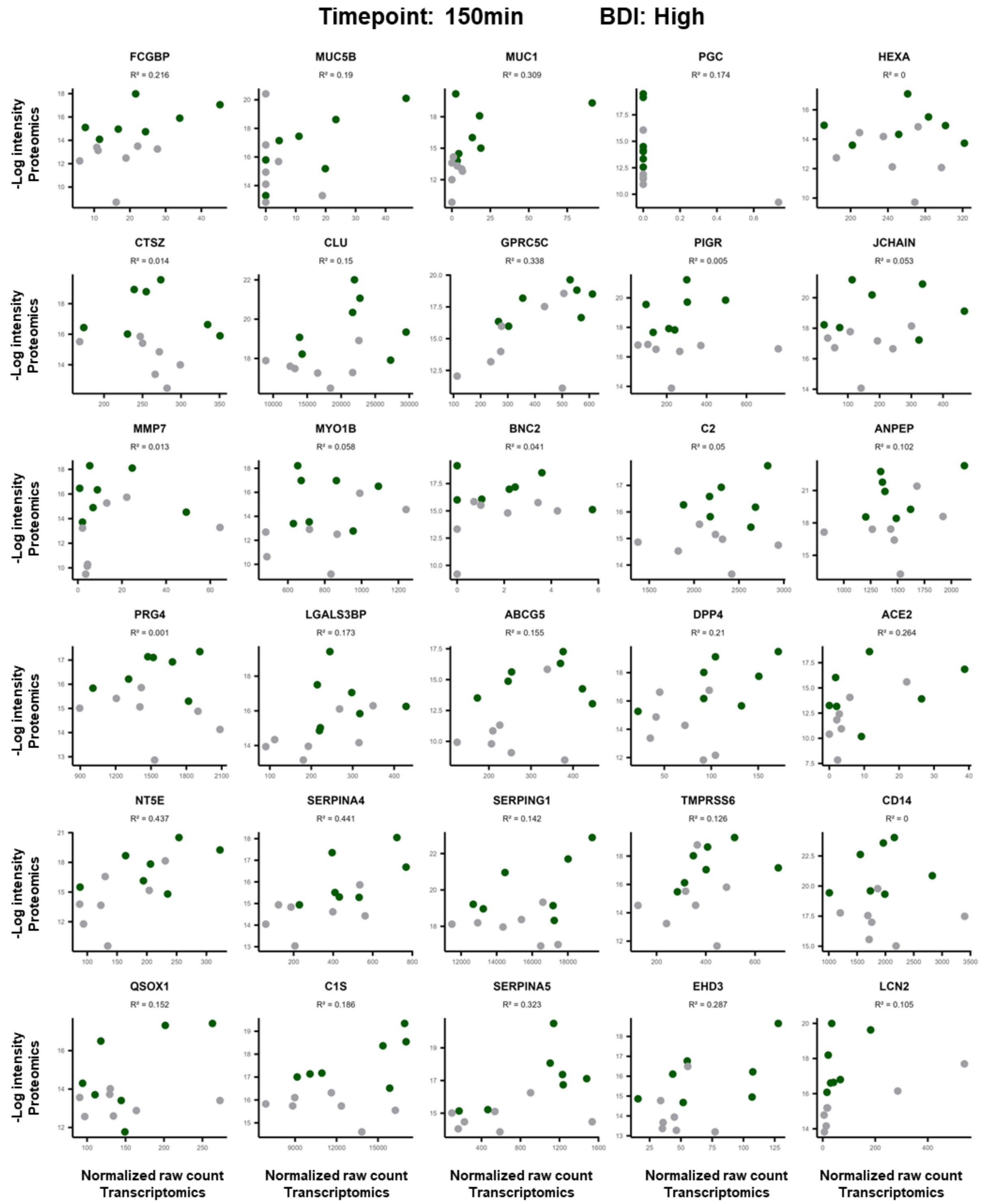

Figure S12. Correlation of RNA sequencing transcripts from baseline biopsies with bile protein intensity in low BDI livers at 150min. Data is presented as protein intensity (-Log transformed, y-axis) correlated with RNA sequencing transcripts (normalized raw counts, x-axis). R-squared values are displayed for each plot. Livers with high biliary viability score are in green, low are in grey.

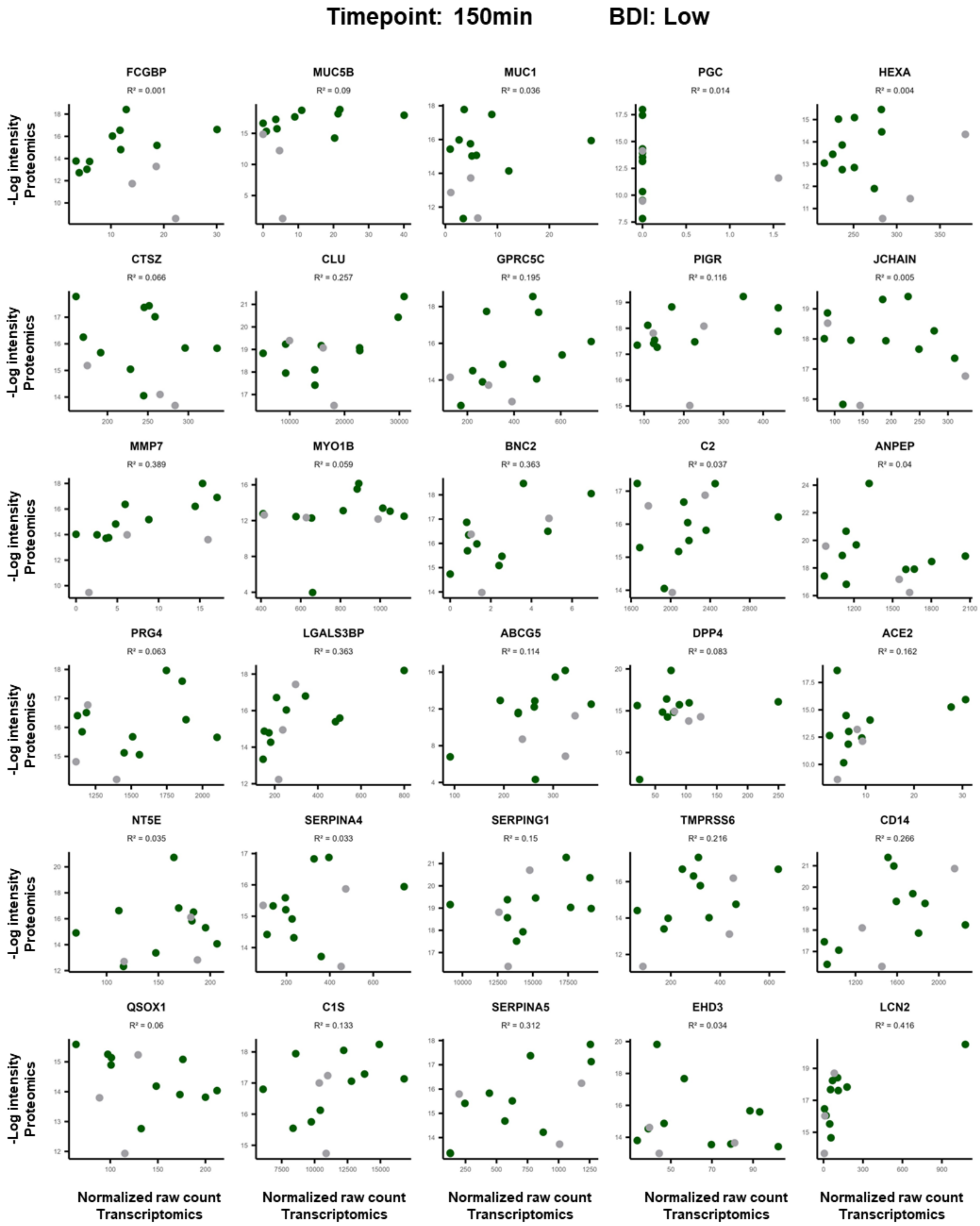

Figure S13. Correlation of RNA sequencing transcripts from baseline biopsies with bile protein intensity in low BDI livers at 150min. Data is presented as protein intensity (-Log transformed, y-axis) correlated with RNA sequencing transcripts (normalized raw counts, x-axis). R-squared values are displayed for each plot. Livers with high biliary viability score are in green, low are in grey.

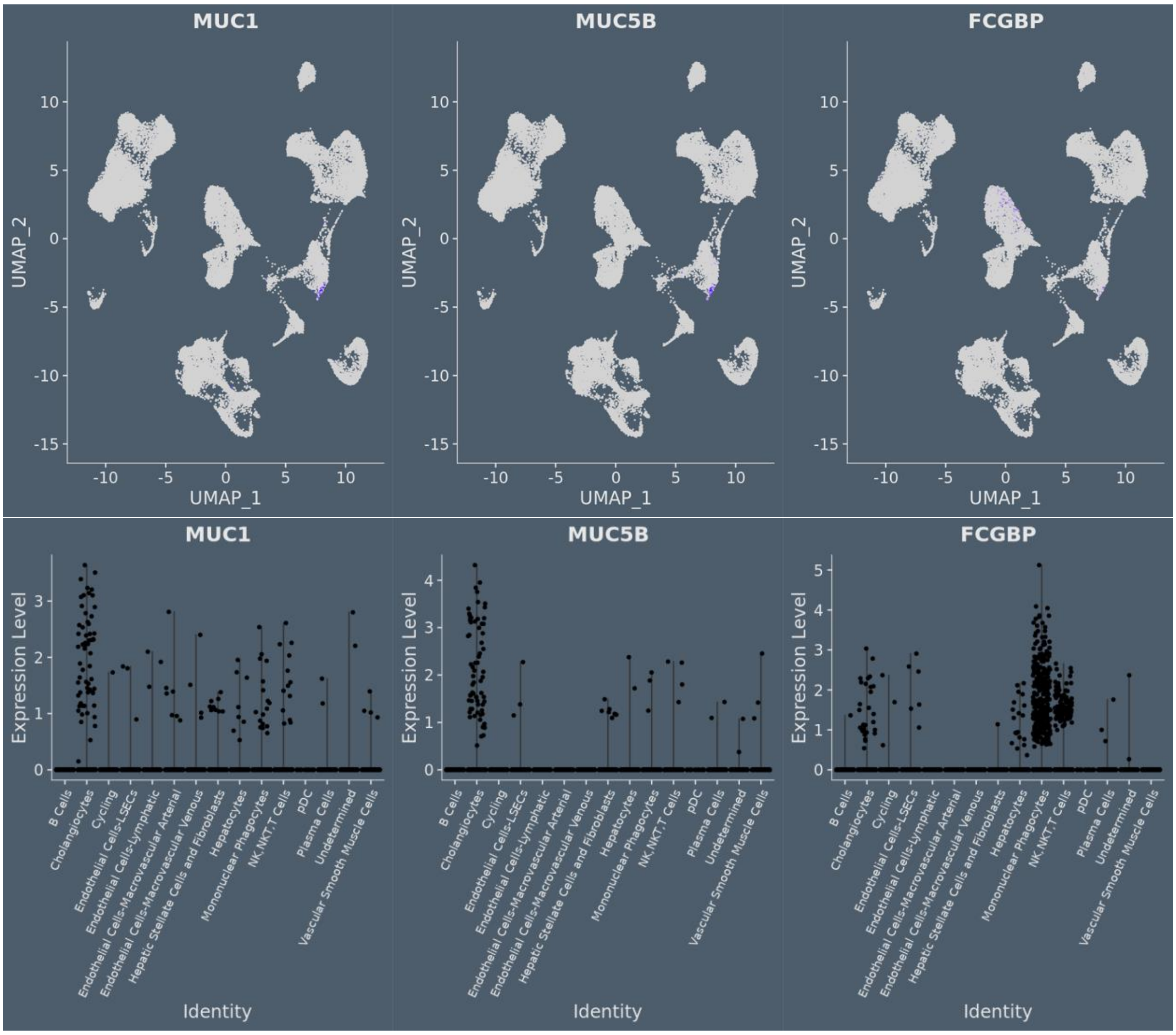

Figure S14. Single-cell RNA sequencing data of MUC1, MUC5B and FCGBP in healthy human livers. Visualization of gene expression across multiple cell types (localization in top panel, in purple; expression level in bottom panel). MUC1 and MUC5B show minimal expression in a variety of cell types, including hepatocytes. However, their main expression lies in cholangiocytes. While the same is true for FCGBP, its predominant expression lies within mononuclear phagocytes. All three described proteins are involved in the initiation and regulation of regenerative processed within the biliary epithelium, including endothelial mesenchymal transition. Data displayed was obtained from a publicly available RNA sequencing data set [1], based on 5 independent studies [2-6].

Table S1. Individual data of donor characteristics for included livers.

Liver number	Donor age	Donor BMI	Gender	Cause of death	Donor type	Time from withdrawal of life support to circulatory arrest (min)	Time from circulatory arrest to cold perfusion (min)	Functional donor warm ischemia time* (min)	Last sodium (mmol/L)	Last AST (U/L)	Last ALT (U/L)	Last GGT (U/L)	Last ALP (U/L)	Hepatectomy time (min)	Static cold ischemia time (min)	DRI (Feng)
1	70-79	24	Female	CVA	DCD	22	17	35	148	27	19	21	71	31	271	3.1960
2	60-69	24	Male	Anoxia	DCD	14	18	28	146	120	60	71	93	31	288	2.9610
3	60-69	24	Male	Anoxia	DCD	13	15	26	139	80	37	115	106	33	305	2.8650
4	50-59	26	Male	Trauma	DCD	9	13	20	139	241	223	18	90	27	296	2.4340
5	60-69	17	Female	CVA	DCD	20	17	31	140	27	15	9	52	42	270	3.3560
6	70-79	25	Male	Anoxia	DCD	15	20	33	136	146	48	20	46	40	295	3.2190
7	40-49	26	Male	Other	DCD	20	18	35	151	31	104	226	136	39	257	2.4450
9	60-69	24	Female	Trauma	DCD	20	15	27	145	28	24	90	123	36	279	2.5260
10	60-69	32	Male	CVA	DCD	16	19	34	142	50	76	80	64	60	230	2.7340
11	50-59	27	Female	Euthanasia	DCD	14	15	27	138	31	50	75	153	53	528	2.5970
12	60-69	24	Male	CVA	DCD	21	14	31	142	25	18	6	56	41	296	3.3560
13	70-79	24	Male	CVA	DCD	16	17	31	141	31	23	17	70	33	274	3.3710
14	50-59	30	Female	CVA	DCD	77	18	34	139	15	11	9	44	31	223	2.5470
15	70-79	26	Female	Trauma	DCD	8	16	22	161	222	62	200	222	45	268	2.7650
16	60-69	25	Male	CVA	DCD	13	18	30	143	16	13	36	46	28	177	2.9210
17	70-79	32	Male	CVA	DCD	21	19	32	139	31	16	29	62	47	357	3.5070
18	60-69	26	Male	Trauma	DCD	27	15	-	144	34	18	10	39	32	248	2.6640
19	60-69	24	Female	CVA	DCD	19	11	32	145	86	18	16	74	44	237	2.9210
20	60-69	26	Male	Anoxia	DCD	11	16	26	138	84	116	102	51	42	288	2.5090
21	60-69	27	Female	CVA	DCD	16	15	28	155	28	25	19	81	40	301	3.4010
22	60-69	27	Male	CVA	DCD	9	14	25	137	20	14	60	47	49	305	2.7340
23	70-79	30	Male	Anoxia	DCD	14	19	32	146	134	144	72	64	39	320	3.1560
24	50-59	44	Female	CVA	DCD	14	17	29	146	39	15	26	67	40	189	2.5470
25	60-69	29	Male	Trauma	DCD	18	16	30	144	18	10	12	44	39	257	2.4770
26	70-79	20	Female	Trauma	DCD	16	18	31	151	44	23	16	71	30	269	3.1770
27	70-79	25	Male	CVA	DCD	8	16	22	146	15	8	16	83	33	123	2.8780
28	60-69	31	Male	Anoxia	DCD	8	19	25	145	24	35	130	59	57	262	2.4750
29	60-69	28	Male	CVA	DCD	58	17	73	140	34	13	10	75	26	282	2.8630
30	60-69	22	Female	Trauma	DCD	30	14	36	149	26	14	47	66	26	283	2.5600
31	50-59	23	Male	Anoxia	DCD	76	16	91	140	137	149	695	589	32	257	2.6510
32	70-79	36	Female	CVA	DCD	15	19	32	149	24	33	43	87	31	166	3.0930
33	60-69	21	Female	Anoxia	DCD	10	15	32	137	82	68	32	65	39	134	2.4940
34	70-79	32	Male	CVA	DCD	15	19	26	143	16	16	21	-	37	289	3.6730
35	70-79	27	Male	Euthanasia	DCD	17	16	20	143	20	30	46	105	53	247	3.8190
36	50-59	29	Male	Anoxia	DCD	16	32	27	144	98	162	103	61	40	272	2.4980
37	60-69	25	Male	Euthanasia	DCD	7	19	22	141	37	46	120	78	37	196	3.4440
38	40-49	36	Male	Trauma	DCD	60	14	65	139	26	52	152	154	40	127	1.7590
39	50-59	26	Male	CVA	DCD	48	17	55	138	21	18	41	69	33	262	2.7400
40	60-69	29	Female	Anoxia	DCD	25	19	42	140	73	61	23	65	36	232	3.1840

41	60-69	24	Male	Anoxia	DCD	15	18	30	137	132	115	16	68	42	179	2.5590
42	60-69	24	Male	Trauma	DCD	10	13	21	139	84	106	133	117	29	155	2.3960
43	60-69	27	Male	CVA	DCD	28	17	27	152	29	19	30	79	37	329	3.4010
44	60-69	26	Male	Anoxia	DCD	13	20	32	143	41	40	100	58	57	194	2.7700
45	60-69	26	Male	Anoxia	DCD	40	15	49	137	131	31	43	40	40	248	3.1840
46	60-69	23	Female	Anoxia	DCD	3	12	15	136	41	160	67	95	41	222	3.1840
47	30-39	25	Female	Other	DCD	48	21	46	139	58	40	114	78	39	158	1.8130
48	60-69	25	Male	Other	DCD	10	16	26	133	74	173	105	58	46	305	3.2030
49	70-79	25	Male	CVA	DCD	43	13	56	139	45	23	23	29	28	285	3.5300
50	60-69	25	Male	Trauma	DCD	75	10	90	149	40	16	41	81	40	154	2.1150
51	60-69	29	Male	Other	DCD	17	12	29	149	120	126	23	74	39	242	3.4210
52	60-69	14	Male	Other	DCD	34	16	-	146	35	24	33	98	35	236	2.7320
53	60-69	24	Male	CVA	DCD	14	14	-	145	61	31	6	35	36	255	3.0000
54	60-69	27	Male	CVA	DCD	20	16	-	144	27	23	27	46	41	247	3.0200
55	70-79	16	Male	Anoxia	DCD	20	13	-	143	118	29	260	50	18	213	3.3710
56	60-69	28	Female	CVA	DCD	26	15	-	142	23	20	23	115	35	324	3.2900

#Validated scoring tool to assess the risk of liver graft failure. * Time from donor saturation <80% or mean arterial pressure <60 mm/Hg to initiation of in-situ cold flushing in the donor. Static cold ischemia time was defined as the time between initiation of cold flushing in the donor and start of DHOPE. Abbreviations: ALP; alkaline phosphatase, ALT; alanine aminotransferase, AST; aspartate aminotransferase, DBD; donation after brain death, DCD; donation after circulatory death, DRI; donor risk index, DRI; Donor risk index, GGT; gamma glutamyl transferase.

Table S2. Viability criteria for determining liver viability at 150mins NMP.

	Parameter	Green zone	Orange zone	Red zone
Hepatocytes	Bile production (mL)	≥ 10*	5 to 10	<5
	Perfusate lactate (mmol/L)	< 1.7	1.7 to 4.0	> 4.0
	Perfusate pH	7.35 – 7.45	7.25 to 7.35	< 7.25
Cholangiocytes	Bile pH	> 7.45	7.40 to 7.45	< 7.40
	ΔpH	> 0.10	0.05 to 0.10	< 0.05
	ΔHCO_3^- (mmol/L)	> 5.0	3.0 to 5.0	< 3.0
	ΔGlucose (mmol/L)	< -5.0	-3.0 to -5.0	> -3.0
Score		2	1	0

Viability criteria that needed to be reached within 150min of normothermic machine perfusion. The green zone includes the four original viability criteria (perfusate pH, lactate, bile production and bile pH) that had to be reached at any time point within 150min after initiation of NMP. The other criteria were secondary criteria that emerged with increasing experience. Orange zone represents potentially acceptable values which are 'on the border', and that could be accepted if the other viability criteria are 'green'. Red zone indicates values that do not meet the viability criteria [7,8].

* Of which ≥ 4 mL in the last hour. Δ indicates the bile value minus the perfusate value. Abbreviations: NMP; normothermic machine perfusion.

Table S3. Blood gas data for included livers.

	Hepatocellular Viability			Cholangiocellular Viability				Outcome		
Liver Number	Lactate	pH	Bile production	pH	Δ pH	Δ HCO3	Δ Glucose	Transplant	Biliary viability score	Biliary viability score group
19	1.1	7.410	Yes	7.660	0.250	15.0	-14.0	Yes	8	High
7	1.2	7.360	Yes	7.490	0.130	6.0	-14.2	Yes	8	High
9	0.1	7.417	Yes	7.565	0.148	9.5	-13.9	Yes	8	High
10	1.2	7.404	Yes	7.675	0.271	10.6	-8.3	Yes	8	High
2	0.2	7.408	Yes	7.547	0.139	9.5	-7.7	Yes	8	High
31	0.4	7.375	Yes	7.536	0.161	10.4	-11.5	Yes	8	High
25	0.5	7.374	Yes	7.547	0.173	9.3	-14.2	Yes	8	High
20	1.8	7.423	Yes	7.580	0.157	6.7	-5.1	Yes	8	High
3	0.2	7.347	Yes	7.465	0.118	8.2	-8.4	Yes	8	High
27	0.0	7.377	Yes	7.487	0.110	10.1	-11.2	Yes	8	High
38	0.4	7.327	Yes	7.614	0.287	15.4	-11.9	Yes	8	High
33	0.3	7.349	Yes	7.878	0.529	49.5	-12.5	Yes	8	High
36	0.2	7.370	Yes	7.523	0.153	8.4	-8.3	Yes	8	High
37	0.8	7.400	Yes	7.522	0.122	8.8	-14.3	Yes	8	High
46	0.7	7.396	Yes	7.542	0.146	6.5	-6.2	Yes	8	High
32	2.2	7.456	Yes	7.789	0.333	34.8	-21.2	Yes	8	High
42	0.1	7.400	Yes	7.500	0.100	6.0	-6.7	Yes	8	High
41	0.1	7.429	Yes	7.811	0.382	43.0	-11.0	Yes	8	High
48	1.2	7.403	Yes	7.543	0.140	15.1	-7.8	Yes	8	High
55	0.1	7.332	Yes	7.559	0.227	14.9	-18.0	Yes	8	High
18	0.4	7.432	Yes	7.484	0.052	7.2	-8.3	Yes	7	High
28	0.1	7.367	Yes	7.524	0.157	9.9	-5.0	Yes	7	High
21	0.5	7.423	Yes	7.503	0.080	8.9	-8.3	Yes	7	High
45	0.5	7.412	Yes	7.503	0.091	9.2	-8.5	Yes	7	High
44	0.1	7.388	Yes	7.470	0.082	7.1	-5.9	Yes	7	High
50	0.1	7.419	Yes	7.572	0.153	21.1	-4.7	Yes	7	High
51	0.3	7.462	Yes	7.515	0.053	15.9	-11.9	Yes	7	High
4	1.1	7.278	Yes	7.423	0.145	7.6	-3.1	Yes	6	High
16	0.3	7.387	Yes	7.458	0.071	5.2	-5.0	Yes	6	High
29	0.4	7.411	Yes	7.506	0.095	3.8	-5.6	Yes	6	High
24	0.8	7.363	Yes	7.455	0.092	7.9	-4.4	Yes	6	High
6	0.1	7.430	Yes	7.439	0.009	7.2	-5.4	Yes	5	High
40	0.7	7.366	Yes	7.427	0.061	4.6	-5.8	Yes	5	High
35	1.0	7.409	Yes	7.440	0.031	4.7	-9.2	Yes	4	Low
22	0.1	7.394	Yes	7.405	0.011	3.9	-3.7	Yes	3	Low
26	3.4	7.360	Yes	7.650	0.290	19.0	-15.4	No	8	High
47	13.9	7.225	Yes	7.338	0.113	5.3	-1.8	No	4	Low
49	0.5	7.360	Yes	7.490	0.130	0.0	-2.2	No	4	Low
23	0.9	7.411	Yes	7.431	0.020	3.3	-3.4	No	3	Low
1	1.5	7.423	Yes	7.380	-0.043	3.6	-7.6	No	3	Low
39	1.0	7.395	Yes	7.426	0.031	1.4	-6.1	No	3	Low
52	0.3	7.400	Yes	7.420	0.020	4.0	-4.3	No	3	Low
13	0.4	7.378	Yes	7.427	0.049	2.3	-3.3	No	2	Low
5	0.8	7.440	Yes	7.400	-0.040	1.0	-1.4	No	1	Low

12	0.2	7.340	Yes	7.360	0.020	2.0	-4.0	No	1	Low
15	0.6	7.424	Yes	7.422	-0.002	1.5	-1.4	No	1	Low
14	1.7	7.400	Yes	7.420	0.020	2.0	3.9	No	1	Low
30	1.4	7.390	Yes	7.425	0.035	1.0	-2.1	No	1	Low
53	0.7	7.401	Yes	7.416	0.015	-9.4	5.3	No	1	Low
11	1.2	7.280	Yes	7.230	-0.050	-1.0	-0.2	No	0	Low
17	0.7	7.390	Yes	7.330	-0.060	2.0	-0.7	No	0	Low
34	3.4	7.384	Yes	7.387	0.003	-1.1	1.0	No	0	Low
43	0.9	7.380	Yes	7.320	-0.060	-1.0	2.8	No	0	Low
54	0.5	7.507	Yes	7.296	-0.211	0.6	2.0	No	0	Low
56	1.7	7.349	Yes	7.339	-0.010	0.9	-0.3	No	0	Low

Numbers represent the blood gas values measured at 150 min NMP. Green indicated that the viability criteria were met. Orange represents that, although the value did not reach the viability criteria, transplantation may still be considered dependent on other values. Red indicates the value did not reach the viability criteria. Biliary viability score was calculated by totaling the assigned values of 2, 1 and 0 for green, orange and red, respectively.

Table S4. Individual area under the curve values for the top 30 highest AUC proteins identified in livers with high biliary viability.

			BDI High		BDI Low		
Gene name	30min	150min	30min	150min	30min	150min	Average AUC
FCGBP	0.84	0.88	0.94	0.99	0.82	0.85	0.90
MUC5B	0.81	0.84	0.84	0.78	0.92	0.92	0.86
MUC1	0.76	0.91	0.73	0.92	0.88	0.88	0.85
PGC	0.82	0.82	0.82	0.93	0.82	0.78	0.84
HEXA	0.81	0.74	0.83	0.80	0.84	0.87	0.84
CTSZ	0.74	0.79	0.74	0.87	0.81	0.88	0.83
CLU	0.78	0.80	0.76	0.90	0.82	0.80	0.82
GPRC5C	0.75	0.78	0.91	0.82	0.72	0.82	0.82
PIGR	0.76	0.87	0.87	0.95	0.66	0.78	0.81
JCHAIN	0.74	0.81	0.79	0.94	0.72	0.80	0.81
MMP7	0.84	0.84	0.86	0.88	0.79	0.71	0.81
MYO1B	0.73	0.75	0.86	0.80	0.71	0.84	0.80
C2	0.80	0.78	0.90	0.90	0.66	0.74	0.80
ANPEP	0.75	0.76	0.81	0.81	0.74	0.81	0.79
PRG4	0.72	0.76	0.85	0.84	0.71	0.75	0.79
LGALS3BP	0.80	0.73	0.85	0.85	0.77	0.68	0.79
ABCG5	0.68	0.75	0.81	0.87	0.68	0.78	0.78
DPP4	0.76	0.75	0.86	0.82	0.74	0.71	0.78
ACE2	0.73	0.81	0.79	0.78	0.73	0.81	0.78
NT5E	0.71	0.76	0.83	0.80	0.72	0.76	0.78
SERPINA4	0.72	0.75	0.73	0.82	0.76	0.80	0.78
BNC2	0.77	0.73	0.82	0.85	0.81	0.62	0.77
SERPING1	0.73	0.75	0.75	0.82	0.73	0.78	0.77
TMPRSS6	0.68	0.73	0.85	0.79	0.63	0.81	0.77
CD14	0.73	0.76	0.83	0.85	0.64	0.75	0.77
QSOX1	0.80	0.77	0.71	0.69	0.88	0.80	0.77
C1S	0.70	0.78	0.85	0.83	0.61	0.79	0.77
SERPINA5	0.74	0.75	0.74	0.82	0.74	0.76	0.77
EHD3	0.70	0.76	0.77	0.81	0.66	0.81	0.76
LCN2	0.81	0.82	0.79	0.88	0.67	0.71	0.76

Average AUC is the mean AUC across BDI high; 30min, 150min, and BDI low; 30min, 150min.

Abbreviations: AUC; area under the curve, BDI; Bile duct injury.

References

1. Brancale, J. & Vilarinho, S. A single cell gene expression atlas of 28 human livers. *J Hepatol* **75**, 219-220 (2021).
2. MacParland, S.A.*, et al.* Single cell RNA sequencing of human liver reveals distinct intrahepatic macrophage populations. *Nat Commun* **9**, 4383 (2018).
3. Aizarani, N.*, et al.* A human liver cell atlas reveals heterogeneity and epithelial progenitors. *Nature* **572**, 199-204 (2019).
4. Segal, J.M.*, et al.* Single cell analysis of human foetal liver captures the transcriptional profile of hepatobiliary hybrid progenitors. *Nat Commun* **10**, 3350 (2019).
5. Tamburini, B.A.J.*, et al.* Chronic Liver Disease in Humans Causes Expansion and Differentiation of Liver Lymphatic Endothelial Cells. *Front Immunol* **10**, 1036 (2019).
6. Ramachandran, P.*, et al.* Resolving the fibrotic niche of human liver cirrhosis at single-cell level. *Nature* **575**, 512-518 (2019).
7. van Leeuwen, O.B.*, et al.* Sequential hypothermic and normothermic machine perfusion enables safe transplantation of high-risk donor livers. *Am J Transplant* **22**, 1658-1670 (2022).
8. van Leeuwen, O.B., de Vries, Y., de Meijer, V.E. & Porte, R.J. Hypothermic machine perfusion before viability testing of previously discarded human livers. *Nat Commun* **12**, 1008 (2021).
