# Supplementary material for: Bile proteome reveals biliary regeneration during normothermic preservation of human donor livers
Source: Nat Commun. 2023 Nov 30;14:7880. doi: 10.1038/s41467-023-43368-y (PMC10689461; doi:10.1038/s41467-023-43368-y)
Supplement: Supplementary file 3 — Reporting Summary [file 41467_2023_43368_MOESM3_ESM.pdf]

nature portfolio

Corresponding author(s): Vincent E de Meijer

Last updated by author(s): Oct 27, 2023

Reporting Summary

Nature Portfolio wishes to improve the reproducibility of the work that we publish. This form provides structure for consistency and transparency in reporting. For further information on Nature Portfolio policies, see our Editorial Policies and the Editorial Policy Checklist.

Statistics

For all statistical analyses, confirm that the following items are present in the figure legend, table legend, main text, or Methods section.

n/a	Confirmed	
☐	☒	The exact sample size (*n*) for each experimental group/condition, given as a discrete number and unit of measurement
☐	☒	A statement on whether measurements were taken from distinct samples or whether the same sample was measured repeatedly
☐	☒	The statistical test(s) used AND whether they are one- or two-sided<br>*Only common tests should be described solely by name; describe more complex techniques in the Methods section.*
☐	☒	A description of all covariates tested
☐	☒	A description of any assumptions or corrections, such as tests of normality and adjustment for multiple comparisons
☐	☒	A full description of the statistical parameters including central tendency (e.g. means) or other basic estimates (e.g. regression coefficient) AND variation (e.g. standard deviation) or associated estimates of uncertainty (e.g. confidence intervals)
☐	☒	For null hypothesis testing, the test statistic (e.g. *F*, *t*, *r*) with confidence intervals, effect sizes, degrees of freedom and *P* value noted<br>*Give P values as exact values whenever suitable.*
☒	☐	For Bayesian analysis, information on the choice of priors and Markov chain Monte Carlo settings
☒	☐	For hierarchical and complex designs, identification of the appropriate level for tests and full reporting of outcomes
☒	☐	Estimates of effect sizes (e.g. Cohen's *d*, Pearson's *r*), indicating how they were calculated

Our web collection on statistics for biologists contains articles on many of the points above.

Software and code

Policy information about availability of computer code

Data collection	No software was used
Data analysis	Perseus (v2.0.10.0) and GraphPad Prism 9 was used for proteomics data analysis. R (v 4.3.1) was used for proteomics data visualisation (heat maps) and for RNA sequencing data analysis, the details of which are specified in the methods section of the manuscript.

For manuscripts utilizing custom algorithms or software that are central to the research but not yet described in published literature, software must be made available to editors and reviewers. We strongly encourage code deposition in a community repository (e.g. GitHub). See the Nature Portfolio guidelines for submitting code & software for further information.

Data

Policy information about availability of data

All manuscripts must include a data availability statement. This statement should provide the following information, where applicable:

- Accession codes, unique identifiers, or web links for publicly available datasets
- A description of any restrictions on data availability
- For clinical datasets or third party data, please ensure that the statement adheres to our policy

Raw mass spectrometry data used for proteomic analysis are available through ProteomeXchange (PRIDE; identifier PXD046355). RNA sequencing data used for transcriptomics analysis are available through the European Nucleotide Archive (identifier E-MTAB-13501). All other data are available in the main text or supplementary materials.

Research involving human participants, their data, or biological material

Policy information about studies with human participants or human data. See also policy information about sex, gender (identity/presentation), and sexual orientation and race, ethnicity and racism.

Reporting on sex and gender	Donor sex is reported in the donor characteristic tables in the main text and supplementary materials. Sex and gender was not used in the study design and has no influence on the donor livers accepted, tested and analyzed in this study.
Reporting on race, ethnicity, or other socially relevant groupings	Race, ethnicity etc was not collected nor relevant to the study. All donor and recipient characteristics are presented in the respective tables in the main text and supplementary materials.
Population characteristics	Human donor livers from deceased donors were used in this study. All livers were initially declined for transplantation nationwide, and were then functionally assessed before either transplanting, or discarding.
Recruitment	Donor livers were recruited based on the patients consent for organ donation. If the liver was declined by all centers nationally, the liver was functionally assessed at our center. This research complies with all relevant ethical regulations. For the current study, recipient data were extracted from the TransplantLines Biobank and Cohort Study of the University Medical Center Groningen (ClinicalTrials.gov Identifier: NCT03272841). The TransplantLines study protocol was approved by the institutional review board (protocol 2014/077) of the University Medical Center Groningen. Written Iinformed consent from all participants was previously obtained.
Ethics oversight	University Medical Center Groningen

Note that full information on the approval of the study protocol must also be provided in the manuscript.

Field-specific reporting

Please select the one below that is the best fit for your research. If you are not sure, read the appropriate sections before making your selection.

- [x] Life sciences
- [ ] Behavioural & social sciences
- [ ] Ecological, evolutionary & environmental sciences

For a reference copy of the document with all sections, see nature.com/documents/nr-reporting-summary-flat.pdf

Life sciences study design

All studies must disclose on these points even when the disclosure is negative.

Sample size	No sample size calculation was performed. Sample size was determined by the number of clinical normothermic liver perfusions performed at our center, from the start of our program to the start of our mass spectrometry analyses (30th March 2019 - 1st July 2022), and where samples at the time points chosen for this study were available. This constituted 55 livers, with a total of 143 bile samples.
Data exclusions	One sample was excluded from the analysis due to no measurable protein present in the sample.
Replication	We did not include replicates, however we did include RNA sequencing to validate proteomic findings, and further confirm these with a publicly available single-cell RNA sequencing data set, which is referenced in the main text of the manuscript and supplementary materials.
Randomization	The order of samples were randomized for mass spectrometry and RNA sequencing analysis.
Blinding	Blinding is not relevant to this study. Livers were tested for viability using predefined criteria, and were not allocated to any group, other than how well the liver fulfilled the viability criteria.

Reporting for specific materials, systems and methods

We require information from authors about some types of materials, experimental systems and methods used in many studies. Here, indicate whether each material, system or method listed is relevant to your study. If you are not sure if a list item applies to your research, read the appropriate section before selecting a response.

Materials & experimental systems

n/a	Involved in the study
☒	☐ Antibodies
☒	☐ Eukaryotic cell lines
☒	☐ Palaeontology and archaeology
☒	☐ Animals and other organisms
☒	☐ Clinical data
☒	☐ Dual use research of concern
☒	☐ Plants

Methods

n/a	Involved in the study
☒	☐ ChIP-seq
☒	☐ Flow cytometry
☒	☐ MRI-based neuroimaging
